# Neural Representation of Associative Threat Learning in Pulvinar Divisions, Lateral Geniculate Nucleus, and Mediodorsal Thalamus in Humans

**DOI:** 10.1101/2025.07.09.663823

**Published:** 2025-07-14

**Authors:** Muhammad Badarnee, Zhenfu Wen, B. Isabel Moallem, Stephen Maren, Mohammed R Milad

**Affiliations:** 1 Department of Psychiatry and Behavioral Sciences, The University of Texas, Health Science Center at Houston, McGovern Medical School, TX USA.; 2 Beckman Institute for Advanced Science and Technology, University of Illinois Urbana-Champaign, Urbana, IL; 3 Department of Psychology, University of Illinois Urbana-Champaign, Champaign, IL

**Keywords:** Thalamus, Pulvinar, Lateral geniculate nucleus, Mediodorsal thalamus, Threat learning, Conditioning, Extinction, Recall extinction, Threat renewal, Emotional memory

## Abstract

Understanding the neural mechanisms underlying associative threat learning is essential for advancing behavioral models of threat and adaptation. We investigated distinct activation patterns across thalamic pulvinar divisions, lateral geniculate nucleus (LGN), and mediodorsal thalamus (MD) during the acquisition of associative threat learning in the MRI. We revealed parallel thalamic learning systems within the anterior pulvinar and MD, supporting distinct mechanisms of automatic survival vs. more deliberate learning. Additionally, our findings support a novel hierarchical pulvinar model during fear conditioning: the medial pulvinar mediates basic threat information from the inferior and lateral divisions to the anterior pulvinar for integrative learning. Pulvinar divisions and MD support extinction learning. These regions also process salience and modulate safe/threat memory expression during extinction recall and threat renewal. The LGN sustains feedforward processing of anticipated visual input throughout all threat phases. This study extends dominant brain models of threat learning and memory, reframing our understanding of distinct thalamic roles in these psychological processes.

## Introduction

Over a century ago, Ivan Pavlov provided foundational behavioral evidence for associative learning, demonstrating that pairing a neutral stimulus with an unconditioned stimulus could elicit a conditioned response(Pavlov, 1904). This discovery laid the groundwork for modern learning theories and has been particularly influential in understanding how humans learn to associate neutral stimuli with threats, a process known as associative threat learning. This survival-oriented learning plays a key role in shaping a broad range of behavioral and emotional responses, such as avoidance, decision-making under threats, and fear regulation(Badarnee et al., 2025; Kolling et al., 2014; Korn and Bach, 2019, 2018; Milad et al., 2013). Research on the neural mechanisms underlying associative threat learning has primarily focused on brain regions involved in emotional regulation, such as the amygdala, hippocampus, and prefrontal cortex (PFC)(Fullana et al., 2015; Krasne et al., 2021; Milad and Quirk, 2012; Vuilleumier et al., 2003). More recently, the thalamus has received growing attention in threat learning(Lithari et al., 2015; Penzo et al., 2015; Ramanathan et al., 2018; Ramanathan and Maren, 2019; Ratigan et al., 2023; Totty et al., 2023), since its role has been reconsidered beyond the traditional view of a mere relay station. Yet, the distinct contribution of this complex structure to associative threat learning remains poorly understood.

The thalamus is a hub structure in the mammalian brain that plays multiple critical roles, ranging from basic sensory processing to higher-order functions and threat learning(Halassa and Sherman, 2019; Hummos et al., 2022; Hwang et al., 2017; Saalmann and Kastner, 2011; Sherman, 2016, 2007). It has long been proposed that threat detection is mediated by the pulvinar and the lateral geniculate nucleus (LGN)(Carr, 2015; Pessoa and Adolphs, 2010; Silverstein and Ingvar, 2015), two major nuclei of the visual thalamus(Arcaro et al., 2015; Casanova and Chalupa, 2023; Takakuwa et al., 2021). The pulvinar projects directly to the amygdala and is believed to subconsciously facilitate rapid survival reactions to threats via a subcortical path (‘low road’)(Carr, 2015; Pessoa and Adolphs, 2010; Rafal et al., 2015; Wei et al., 2015). The LGN, on the other hand, is part of a slower but more accurate neural pathway, connecting peripheral information to cortical regions for comprehensive processing (‘high road’). Additionally, the involvement of the mediodorsal thalamus (MD) in threat learning has also been proposed(Lee and Shin, 2016; Lee et al., 2011). Although this nucleus is not typically classified as part of the visual thalamus, it serves as a high-order region reciprocally connected to the amygdala and PFC while playing a critical role in executive functions(Hwang et al., 2020; Li et al., 2022; Mukherjee et al., 2021; Wolff and Halassa, 2024).

The general implication of these nuclei in threat processing has been demonstrated in primates and rodent models. It has been shown that exposing primates to snake images elicited increased neuronal firing within the pulvinar(Le et al., 2013). Excitation of neurons within the LGN enhanced the acquisition of eyeblink conditioning in rodents(Halverson and Freeman, 2010). Inhibiting this nucleus was associated with impaired conditioned responses(Shi and Davis, 2001; Steinmetz et al., 2013), and activating GABA neurons in the LGN reduced freezing response to an overhead dark shadow that mimics a real-world predator in rats(Salay and Huberman, 2021). Reduced freezing was also observed after MD lesions(Li et al., 2004) and when the connections with the anterior cingulate cortex were ablated(Zheng et al., 2020). Lesions within the MD are also associated with impaired fear extinction(Lee et al., 2011), and injecting gabazine, a modulator of extra-synaptic GABA receptors, into the MD facilitated extinction(Paydar et al., 2014).

Evidence regarding the involvement of the thalamus in the context of threat learning in humans is relatively sparse. Much of our knowledge comes from studies focusing on attention, perception, and decision-making. For example, the LGN has been mainly viewed as a transmitter of peripheral information to other brain regions and found to be associated with selective attention and anticipation of visual stimuli(Mahoney and Schmidt, 2024; O’Connor et al., 2002; Saalmann and Kastner, 2009). The MD contribution to perception, spatial attention, and decision-making has also been reported(Griffiths et al., 2022; Wurtz et al., 2011). Beyond cognitive research, some human studies have specifically investigated the pulvinar’s engagement in threat detection. Individuals with increased fiber density in the pulvinar-amygdala pathway showed an enhanced ability to recognize fearful faces(McFadyen et al., 2019). A patient with a complete lesion in the left pulvinar showed slower responses to threatening images when stimuli were presented on the ipsilesional, but not contralesional, field(Ward et al., 2005). In agreement with this, unseen stimuli (fearful vs. happy faces) presented on the blind side of patients with hemianopia moderated their performance when the pulvinar was spared but not when it was lesioned(Bertini et al., 2018).

**Fig 1.**
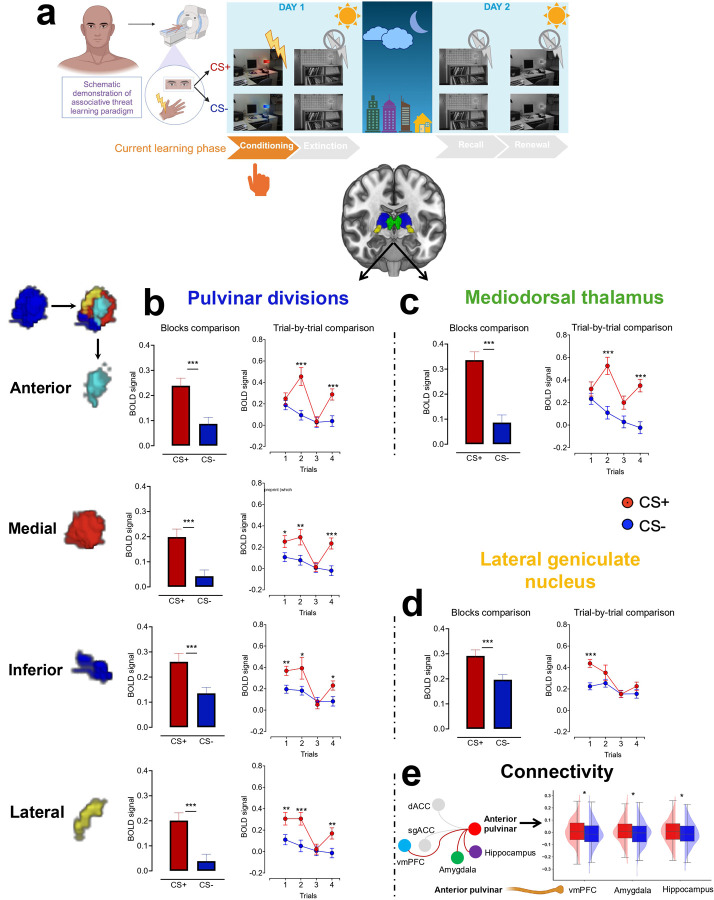
Neural representation of associative threat learning in pulvinar divisions, MD and LGN. **a.** Human fear conditioning paradigm in the fMRI. **b-d.** Means ± SE of activation in response to CS+ vs. CS− at both block-wise and trial-wise levels within the pulvinar divisions (**b**), MD (**c**), and LGN (**d**). **e.** ROI-to-ROI connectivity. Right: the red line represents significant positive connectivity to CS+ vs. CS− (P_FDR_ < 0.05), while the gray lines indicate non-significant connectivity. Thalamic nuclei that showed no significant connectivity with other regions (i.e., amygdala, hippocampus, etc.) were omitted from the visualization. Left: boxplots and kernel density estimates illustrate the distribution of connectivity values in response to CS+ and CS−. SE: Standard error. MD: Mediodorsal thalamus. LGN: Lateral geniculate nucleus. CS+ vs. CS−: Conditioned stimulus predicting shock vs. no shock. ROI: Region of interest. *p<0.05, **p<0.01, ***p<0.001 Display items in panel **a** were created using BioRender (BioRender.com).

Specific thalamic contribution to threat processing is still largely unknown, and translational research on this topic is limited. We aim to address these critical gaps in our understanding by investigating the distinct neural representations of associative threat learning in the human pulvinar divisions, LGN, and MD. We analyzed the neuroimaging data of 293–412 controls. All participants underwent a two-day threat learning paradigm(Milad et al., 2009, 2007; Wen et al., 2024, 2022) while in the fMRI scanner. The conditioned stimulus (CS+) (e.g., red light) was associated with an electric shock (unconditioned stimulus US, 62.5% reinforcement rate), while a control light (e.g., blue) was never paired with the US (CS−). This conditioning phase occurred in a computer-displayed visual context A (e.g., an office). The extinction learning included presenting the CS+ and CS− with no US reinforcement in a distinct context B (e.g., bookcase). In extinction recall and threat renewal, the extinguished stimuli were presented with no US reinforcement within contexts B (safe contextual cues) and A (threat contextual cues), respectively. Schematic illustrations of each phase of the paradigm are presented in Figs. 1a, 4a–6a.

We focus on neural activation patterns within pulvinar divisions, LGN, and MD while acquiring the CS-US association. As associative learning is a rapid psychological process(Konrad et al., 2024), we analyzed the first four trials of all threat learning phases. We compared brain activation (BOLD) to the CS+ vs. CS− at the block level by averaging the activation across all four trials and at a trial-by-trial level to provide finer activation temporal resolution. This dual approach allows us to capture the general neural responses associated with associative threat learning and provides new insights into trial-level dynamics(Wen et al., 2022). The current dominant neurocognitive thalamic models(Sherman, 2007; Sherman and Guillery, 2006) highlight the thalamus’s role in mediating cortical-cortical communications and facilitating high-order functions. Based on this view, we anticipate distinct pulvinar, MD, and LGN roles. The LGN, as a first-order nucleus, is expected to serve relay functions, transmitting information with minimal integrations. The pulvinar and MD, on the other hand, as higher-order nuclei, are likely involved in more complex processing, potentially integrating threat-related information.

## Results

### Parallel functional representation of associative threat learning in the anterior pulvinar and MD during conditioning

The anterior pulvinar and MD contribute to early associative threat learning, as evidenced by increased functional activation in response to CS+ compared to CS− at the block level (Fig. 1b–c). The trial-wise analysis revealed a distinct activation pattern. In the first trial, we observed similar activation levels for both CS+ and CS− (no significant difference), but in the subsequent trial, the BOLD signal in both regions was heightened specifically for CS+ (Fig. 1b–c, Extended Tables 1–2). In our paradigm, the electric shock was paired with CS+ at the end of the CS presentation. The similarity in BOLD responses to CS+ and CS− during the first trial likely reflects an initial equivalence in the emotional valence of the stimuli. The gradual increase in activation for CS+ by the second trial suggests a shift in the emotional valence, indicating rapid associative learning.

**Fig 2.**
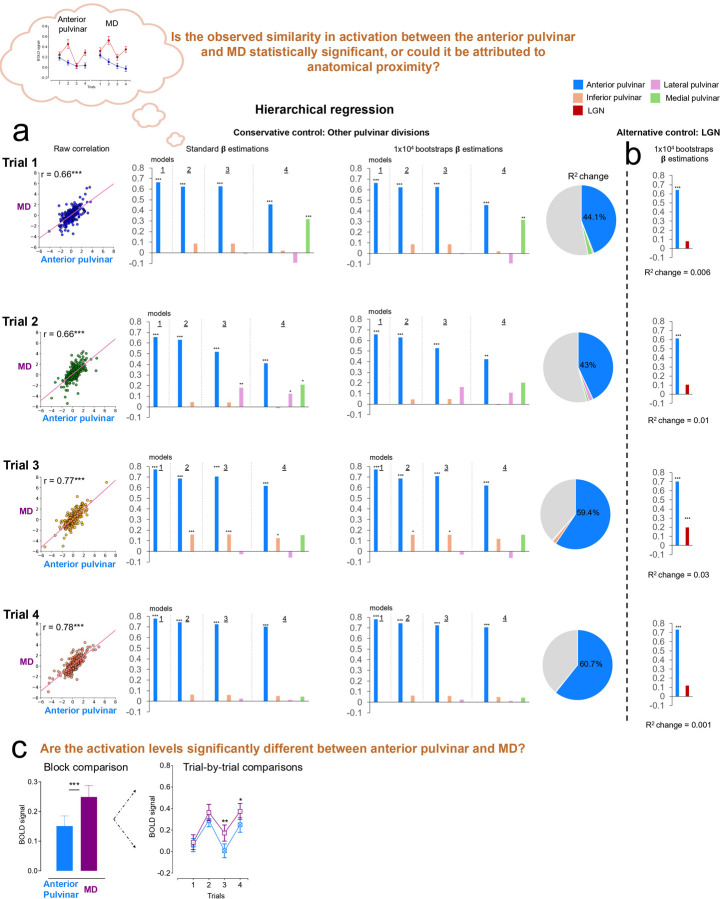
Quantifying the relationships between the anterior pulvinar and MD during conditioning. **a.** Hierarchical regression models of trial-wise relationships between the anterior pulvinar and MD activations while controlling for a potential effect of anatomical proximity. The effect of anatomical proximity was controlled by progressively adding other pulvinar divisions as controls. **b.** Results of a hierarchical model that included the LGN as an alternative control, distinct from the pulvinar. **c.** Comparison of activation levels in the anterior pulvinar and MD at both block-wise and trial-wise levels (means ± SE). *p<0.05, **p<0.01, ***p<0.001 MD: Mediodorsal thalamus. LGN: Lateral geniculate nucleus. SE: Standard error.

The similarity in trial-level activation patterns in the anterior pulvinar and MD raises the question of whether this apparent similarity truly reflects a parallel functional contribution to associative threat learning. To test this possibility, we first quantified the trial-wise relationships using Pearson correlation coefficients between the two regions. The observed correlations support consistent co-activation across all corresponding trials (*r* values ≥ 0.66, p < 0.001, Fig. 2a, left). However, this approach could not exclude the possibility that the shared variance is a consequence of shared anatomical proximity, particularly because both regions are part of the same brain structure, i.e., the thalamus. To control for this confound, we conducted a hierarchical regression model in four steps for each trial. We modeled MD activation as the target and progressively added the pulvinar divisions as covariates. Despite small variations in regression weights (beta values) across control models, the anterior pulvinar consistently exhibited the highest beta estimates (Fig. 2a, middle). Importantly, these values remain consistent across 10,000 bootstrapping iterations with replacements, suggesting that the anterior pulvinar effect is robust and stable across resampled datasets (Fig. 2a, right). Additionally, the anterior pulvinar explained the largest proportion of variance in MD activation (*r*^2^ = 43–60.7%) relative to other pulvinar divisions, which negligibly contributed to this parameter (pie charts, Fig. 2a). Finally, applying the same analytical approach while controlling for the LGN activation as an alternative anatomical control beyond the pulvinar itself, yielded similar results (Fig. 2b). Together, these analyses support preferential trial-wise functional co-activations between the anterior pulvinar and MD, independent of shared anatomical proximity.

Although co-activation does not necessarily imply similar activation magnitude, we further tested the differences in activation levels in the anterior pulvinar and MD. We used a t-test to capture the differences in the overall activation at the block level and repeated measures analysis of variance (RM-ANOVA) to capture the activation differences at the trial level. The results showed that the overall MD activation in response to CS+ was higher than the anterior pulvinar response (p-value < 0.001, Fig. 2c). The trial-wise analysis revealed heightened activation in the MD compared to the anterior pulvinar, particularly during trials 3 and 4 (p_fdr_-values < 0.05, Fig. 2c). The increased activation within the MD potentially reflects its role in deeper processing of threat relatively to the anterior pulvinar.

### A data-driven approach reveals hierarchical functional processing of threat in pulvinar divisions

The anatomical and functional interconnections between pulvinar divisions remain insufficiently characterized. To address this gap and understand the pulvinar’s role in early associative threat learning, we integrated findings from complementary analyses into a novel model designed to investigate the functional relationships between pulvinar divisions.

**Fig 3.**
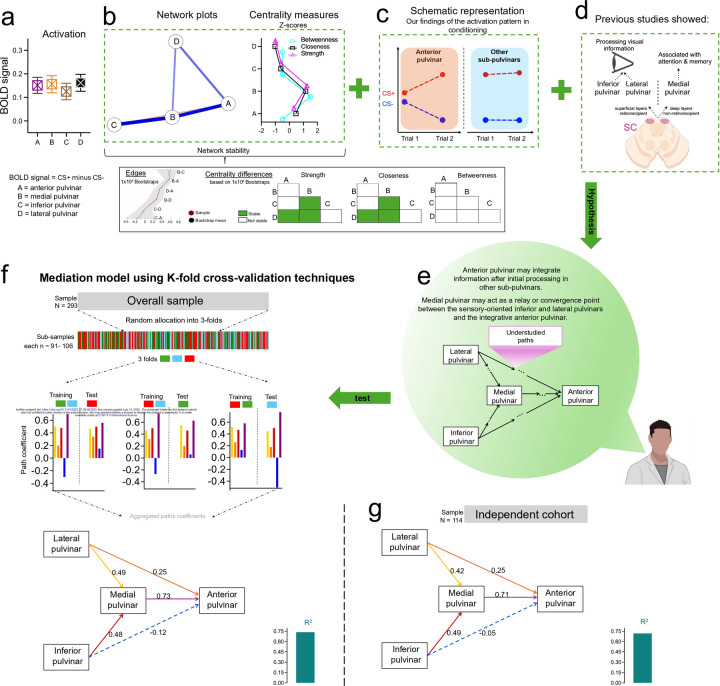
A data-driven approach to understanding the functional relationships between pulvinar divisions during conditioning **a.** Means ± SE of activation differences between pulvinar divisions. **b.** Network analysis reveals that the medial pulvinar serves as a central hub, mediating interactions among pulvinar divisions and exhibiting increased centrality measures. **c.** Schematic visualization of activation patterns we observed in pulvinar divisions. **d.** Previous studies suggest that the inferior and lateral pulvinar are involved in processing basic visual information, while the medial pulvinar is associated with higher-level functions, including working memory. **e.** Based on **b-d**, we hypothesize that the medial pulvinar mediates the relationships with other divisions. **f.** Mediation analysis supports our hypothesis (panel **e**). **g.** Validation of the mediation model on an additional independent sample. Dashed paths in panels **f** and **g** represent statistically unstable paths, while the continuous paths indicate stable paths. SE: Standard error. SC: Superior colliculus. *p<0.05, **p<0.01, ***p<0.001 Display items in panels **d** and **e** were created using BioRender (BioRender.com).

We first defined the activation within each pulvinar division as the differences in BOLD signal at the block level in response to CS+ compared to CS−. We then performed RM-ANOVA to test whether the pulvinar divisions were engaged with different activation levels. We found no activation differences, suggesting similar processing levels of CS+ information across all pulvinar divisions (Fig. 3a). Using network analysis, we explored the underlying functional dynamics between the divisions. We employed the EBICglasso method(Foygel and Drton, 2010) to estimate a sparse Gaussian graphical model. To assess the stability of the network and provide robust estimates of edges and centrality measures, we conducted 10,000 bootstrap resamples. This approach allowed us to compute 95% confidence intervals, ensuring the reliability of our findings. The combined use of EBICglasso and bootstrapping is essential for accurately capturing the dynamics of pulvinar division interactions. The resulting network included four nodes and five edges. The network plot pointed to stable direct edges connecting the lateral pulvinar to the anterior pulvinar and indirect edges connecting both the lateral and inferior pulvinar to the anterior through the medial pulvinar (Fig. 3b). Additionally, the medial pulvinar exhibited the highest centrality values, a measure that captures the node importance within a network, indicating a potential hub role of this division in associative threat learning (Fig. 3b).

These underlying dynamics and the activation timing in the trial-wise analyses (overview in Fig. 3c and details in Fig. 1b) led us to propose a functional model of the relationships between pulvinar divisions. Specifically, the increased activation induced by CS+ during trial 1 in the medial, inferior, and lateral pulvinar suggests that these divisions process CS+ information before the anterior pulvinar, which exhibited a delayed BOLD response starting in trial 2. The medial pulvinar mediates connections within the pulvinar network and is associated with elevated centrality values. Together, these findings highlight the hub role of the medial pulvinar, integrating CS+ information at a higher level, compared to sensory-driven processing in the inferior and lateral pulvinar. This possible hierarchical organization is supported by previous evidence showing that the inferior and lateral pulvinar are associated with processing basic sensory information(Berman and Wurtz, 2010, 2008; Cortes et al., 2024), while the medial pulvinar is implicated in higher-order processing, including attention and working memory(Homman-Ludiye and Bourne, 2019) (Fig. 3d).

This data-driven approach provides a new perspective on the pulvinar divisions’ functional specialization during associative threat learning. We hypothesize that, during this process, the activation in the medial pulvinar mediates the functional relationships between the inferior and lateral divisions with the anterior pulvinar (Fig. 3e). To test this, we conducted a mediation model analysis and evaluated the model robustness using the k-fold cross-validation method. The sample (*N* = 293) was randomly divided into three groups of 91, 96, and 106 subjects. Each sub-sample was used to test the model while the remaining groups served as a training phase. This resulted in six iterations of the mediation model. The 95% Confidence Intervals for each path coefficient were estimated using 10,000 bootstrapping replications. This choice was made to achieve greater precision and stability in the confidence interval estimates. Finally, applying the mediation analysis to another independent cohort (*N* = 114) confirmed the model’s applicability. The results in Figs. 3f–3g support the mediating role of the medial pulvinar, demonstrating significant indirect paths from the lateral and inferior divisions to the anterior pulvinar. Detailed parameters are presented in Extended Table 3.

### Pulvinar divisions and MD support extinction learning while preserving threat memory across contextual cues

**Fig 4.**
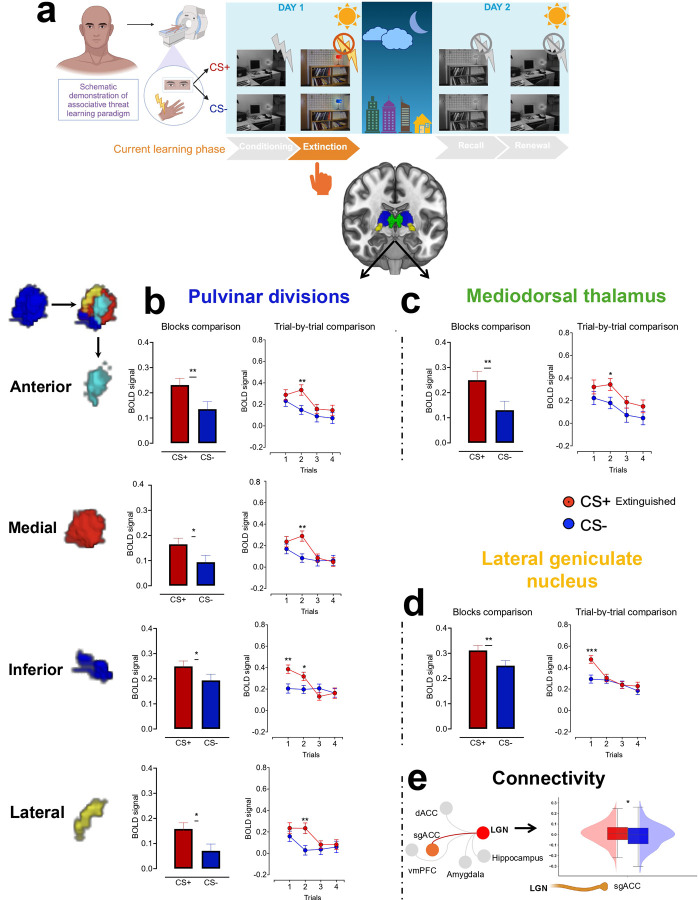
Neural representation of extinction learning in pulvinar divisions, MD and LGN. **a.** Human extinction learning paradigm in the fMRI. **b-d.** Means ± SE of activation in response to extinguished CS+ vs. CS− at both block-wise and trial-wise levels within the pulvinar divisions (**b**), MD (**c**), and LGN (**d**). **e.** ROI-to-ROI connectivity. Right: the red line represents significant positive connectivity to extinguished CS+ vs. CS− (P_FDR_ < 0.05), while the gray lines indicate no significant connectivity. Thalamic nuclei that showed non-significant connectivity with other regions (i.e., amygdala, hippocampus, etc.) were omitted from the visualization. Left: boxplots and kernel density estimates illustrate the distribution of connectivity values in response to extinguished CS+ and CS−. SE: Standard error. MD: Mediodorsal thalamus. LGN: Lateral geniculate nucleus. Extinguished CS+ vs. CS−: A conditioned stimulus that no longer predicts shock vs. a stimulus that was never paired with shock. ROI: Region of interest. *p<0.05, **p<0.01, ***p<0.001 Display items in panel **a** were created using BioRender (BioRender.com).

After our comprehensive examination of the thalamic contribution to threat conditioning, we moved forward to examine the neural contributions of the same regions to extinction learning. During this phase, we found higher activation in response to CS+ than CS− across all pulvinar divisions and MD at the block level. The trial-wise analysis demonstrated that these differences are mainly driven by the first two trials (Figs. 4b and 4c). Together, the pulvinar divisions and MD showed a similar activation pattern where the BOLD signal induced by the CS+ is significantly diminished after trial 2, highlighting these nuclei’s engagement in rapid extinction learning. Detailed parameters are presented in Extended Tables 4–5.

**Fig 5.**
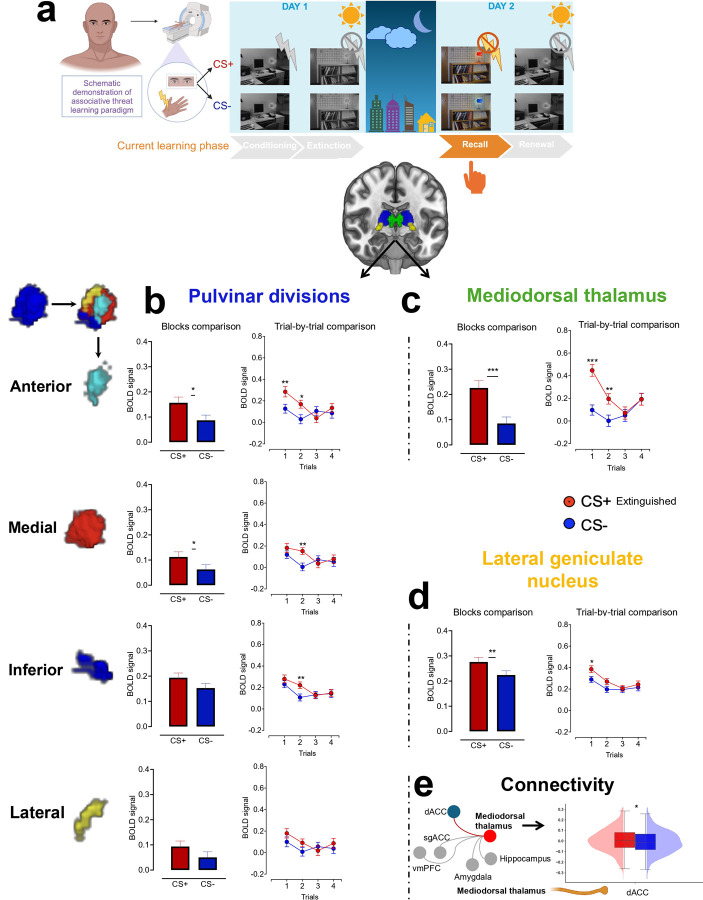
Neural representation of extinction recall in pulvinar divisions, MD and LGN. **a.** Human extinction recall paradigm in the fMRI conducted within safe contextual cues. **b-d.** Means ± SE of activation in response to extinguished CS+ vs. CS− at both block-wise and trial-wise levels within the pulvinar divisions (**b**), MD (**c**), and LGN (**d**). **e.** ROI-to-ROI connectivity. Right: the red line represents significant positive connectivity to extinguished CS+ vs. CS− (P_FDR_ < 0.05), while the gray lines indicate non-significant connectivity. Thalamic nuclei that showed no significant connectivity with other regions (i.e., amygdala, hippocampus, etc.) were omitted from the visualization. Left: boxplots and kernel density estimates illustrate the distribution of connectivity values in response to extinguished CS+ and CS−. SE: Standard error. MD: Mediodorsal thalamus. LGN: Lateral geniculate nucleus. Extinguished CS+ vs. CS−: A conditioned stimulus that no longer predicts shock vs. a stimulus that was never paired with shock. ROI: Region of interest. *p<0.05, **p<0.01, ***p<0.001 Display items in panel **a** were created using BioRender (BioRender.com).

During extinction recall, the anterior pulvinar and MD exhibited similar functional patterns at both block and trial levels, with increased activation to CS+ compared to CS− during the first two trials. In contrast, the medial and inferior pulvinar maintained the BOLD signal to CS+ in the second trial while showing reduced responses to CS−. We observed no activation differences in the lateral pulvinar (Fig. 5b–c, Extended Tables 6–7). This finding highlights the involvement of most pulvinar divisions and MD in sustaining threat memory under safe contextual cues and suggests retrieval suppression of the extinguished threat.

**Fig 6.**
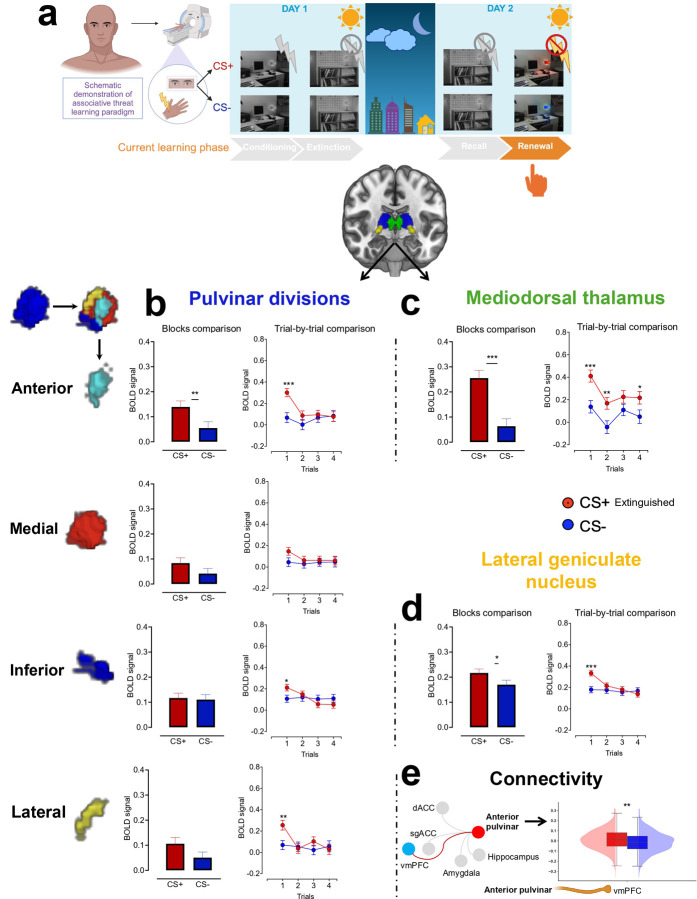
Neural representation of threat renewal in pulvinar divisions, MD and LGN. **a.** Human threat renewal paradigm in the fMRI conducted within threat contextual cues in the original context where fear conditioning occurred. **b-d.** Means ± SE of activation in response to extinguished CS+ vs. CS− at both block-wise and trial-wise levels within the pulvinar divisions (**b**), MD (**c**), and LGN (**d**). **e.** ROI-to-ROI connectivity. Right: the red line represents significant positive connectivity to extinguished CS+ vs. CS− (P_FDR_ < 0.05), while the gray lines indicate non-significant connectivity. Thalamic nuclei that showed no significant connectivity with other regions (i.e., amygdala, hippocampus, etc.) were omitted from the visualization. Left: boxplots and kernel density estimates illustrate the distribution of connectivity values in response to extinguished CS+ and CS−. SE: Standard error. MD: Mediodorsal thalamus. LGN: Lateral geniculate nucleus. Extinguished CS+ vs. CS−: A conditioned stimulus that no longer predicts shock vs. a stimulus that was never paired with shock. ROI: Region of interest. *p<0.05, **p<0.01, ***p<0.001 Display items in panel **a** were created using BioRender (BioRender.com).

Changing the contextual cues to a threat background where the original associative learning occurred elicited increased activation in response to CS+ in the anterior, inferior, and lateral pulvinar divisions along with the MD (Fig. 6b–c, Extended Tables 8–9). These regions are, therefore, involved in the reactivation of threat memory, a hallmark process in the threat renewal phase.

### Consistent LGN activation patterns across all threat learning phases align with principles of feedforward processing.

At the block level, we observed elevated activation in response to CS+ compared to CS− across conditioning, extinction, recall, and renewal. These differences were driven by the first trial, during which an increased BOLD signal in response to CS+ was followed by a decline in subsequent trials (Figs. 1d, 4d–6d, Extended Tables 2, 5, 7, and 9). These findings suggest that the LGN plays a consistent role across different phases of threat learning, potentially reflecting early attentional processing or anticipation of upcoming visual stimuli.

#### Thalamic connectivity underlies threat learning and memory

We tested the relationships between each thalamic nucleus and core brain regions within the ‘fear circuit’(Herry et al., 2010; Maren and Quirk, 2004; Milad et al., 2014; Milad and Quirk, 2012; Tovote et al., 2015) by seeding the nuclei to target the amygdala, hippocampus, ventromedial prefrontal cortex (vmPFC), subgenual anterior cingulate cortex (sgACC), and dorsal anterior cingulate cortex (dACC). During conditioning, the anterior pulvinar exhibited positive connectivity with the amygdala, vmPFC, and hippocampus (all p_fdr_ < 0.05 and all 95% bias-corrected and accelerated (BCa) confidence intervals (CI) of the mean differences from 10,000 bootstrap resamples excluded zero; Cohen’s ds = 0.14, 0.14, and 0.15, with corresponding 95% parametric CIs also excluding zero) (Fig. 1e). The connectivity with the amygdala likely supports encoding the emotional valence of the newly learned CS-US associations, while the engagement of the hippocampus suggests prioritizing the contextual CS+ information. The connectivity with the vmPFC may underlie a process of top-down control release to enhance fear expression or encode threat information for tracking and supporting decision-making in future encounters.

During extinction learning, we found increased LGN-sgACC connectivity (p_fdr_ < 0.05 and 95% BCa CIs of the mean differences from 10,000 bootstrap resamples excluded zero; Cohen’s d = 0.15, 95% parametric CI [0.035, 0.256]), suggesting sgACC involvement in emotional regulation (Fig. 4e). Interestingly, in recall and renewal, we found that the learners, i.e., the anterior pulvinar and MD contribute to supporting either safe or threatening memory, depending on contextual cues. Specifically, the MD-dACC connectivity supported safe memory in recall (p_fdr_ < 0.05 and 95% BCa CI of the mean differences from 10,000 bootstrap resamples excluded zero; Cohen’s d = 0.13, 95% parametric CI [0.031, 0.225]; Fig. 5e), whereas anterior pulvinar-vmPFC supported threatening memory in renewal (p_fdr_ < 0.01 and 95% BCa CI of the mean differences from 10,000 bootstrap resamples excluded zero; Cohen’s d = 0.20, 95% parametric CI [0.084, 0.306]; Fig. 6e).

## Discussion

**Fig 7.**
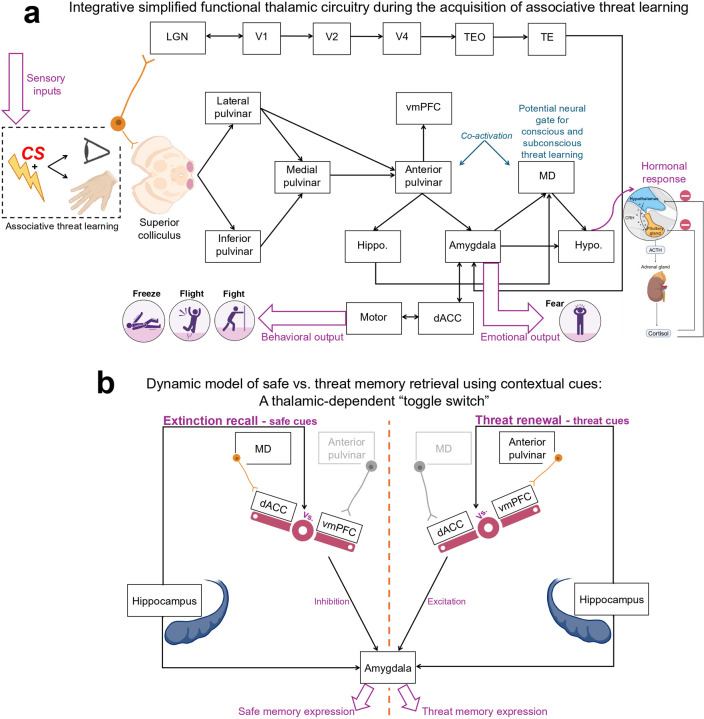
Neurobehavioral models of thalamic involvement in associative threat learning and memory. **a.** Schematic illustration of thalamic circuitry during the acquisition of associative threat learning, highlighting interactions between pulvinar divisions, MD, and LGN with key brain regions involved in fear expression. **b.** A thalamic-dependent “toggle switch” regulates the retrieval of safety vs. threat-related memory. The MD-dACC connectivity modulates the interaction between dACC and vmPFC, promoting vmPFC dominance during extinction recall. In contrast, the anterior pulvinar-vmPFC connectivity promotes dACC dominance, enhancing the expression of threat memory during threat renewal. MD: Mediodorsal thalamus. LGN: Lateral geniculate nucleus. vmPFC = Ventromedial prefrontal cortex. dACC = Dorsal anterior cingulate cortex. V1, V2, and V4: Primary, secondary, and fourth visual areas. TEO, TE: Temporal cortex regions. Hypo.: Hypothalamus. Hippo.: Hippocampus.

We examined the neural representation of associative threat learning within the pulvinar divisions, LGN, and MD, providing new insights into thalamic involvement in this adaptive behavior in humans. We identified distinct roles among these thalamic nuclei with respect to their activation profiles during threat learning and memory. The anterior pulvinar and MD exhibited parallel activation patterns consistent with associative learning, reflecting their roles in automatic survival responses and deliberate threat processing. We propose a novel hierarchical model for processing threat information in the pulvinar divisions. The medial pulvinar mediates basic sensory information from the inferior and lateral divisions to the anterior pulvinar for higher-order integrative learning. Both pulvinar and MD were involved in extinction and showed activation consistent with the salience processing of threat-related memories during extinction recall and threat renewal. The LGN primarily represented feedforward processing, anticipating upcoming visual stimuli throughout all phases of threat learning. We integrated these insights into schematic models underlying the emotional and behavioral expression of threat learning and memory, providing a neural framework for studying related human behaviors (Fig. 7a–b).

The anterior pulvinar and MD co-activation patterns demonstrate a parallel contribution to threat learning. The similar activation in response to CS+ and CS− at the first trial, followed by a heightened activation specific to CS+ in the next trial, is consistent with the acquisition of the CS-US association. The responses’ similarity to both types of CS during the first trial suggests an equivalent initial emotional valence. The gradual increase in the activation, specifically to CS+ by the end of trial 1, suggests a shift in the emotional valence of CS+, indicating rapid associative learning. This parallel specialized role highlights the anterior pulvinar and MD as central thalamic hubs for integrating CS-US information, specifying the two-system model proposed by LeDoux and Pine(LeDoux and Pine, 2016). Briefly, fear processing, according to this model, involves two distinct pathways: a subcortical route for rapid threat detection and species-specific defense reactions and a cortical route for deliberate threat evaluation and conscious fear experience. Learning within the anterior pulvinar appears to support rapid automatic processes of the subcortical pathway. This aligns with previous reports that demonstrated the role of the pulvinar-amygdala pathway in encoding negative emotions in humans(Bridge et al., 2015; Koller et al., 2019; Kragel et al., 2021; McFadyen et al., 2019; Rafal et al., 2015). Indeed, the increased connectivity that we observed with the amygdala, hippocampus, and vmPFC during threat conditioning aligns with this role. Specifically, in this context, the amygdala likely contributes to tagging a negative emotional valence to CS+ and triggering fight, flight, or freeze reactions(Adolphs et al., 1994; Costa et al., 2022; Fanselow and LeDoux, 1999; Phelps and LeDoux, 2005; Wen et al., 2024, 2022). The connectivity with the hippocampus likely facilitates contextual encoding of the environmental characteristics of the aversive event(Maren, 2001; Maren et al., 2013). In turn, the connectivity with vmPFC appears to support salience encoding for tracking future encounters with the learned threat(Battaglia et al., 2020) and facilitating decision-making and emotion regulation(Milad and Quirk, 2012, 2002; Nejati et al., 2021).

On the other hand, MD activation aligns more closely with the cortical pathway, facilitating conscious and deliberate threat encoding. This is supported by our findings, which showed increased MD activation in response to CS+, compared to the anterior pulvinar during threat learning, indicating a broader or deeper processing of the CS-US association. Additionally, this interpretation is supported by substantial evidence pointing to well-established anatomical pathways connecting the MD with the PFC and demonstrating its role in decision-making and learning(Behrens et al., 2003; Hwang et al., 2020; Li et al., 2022; Wolff and Halassa, 2024). This is along with reports that underscored the contribution of the MD-cortical loops to supporting a conscious experience in humans(Griffiths et al., 2022; Whyte et al., 2024). Together, these findings advance our understanding of the two-system model of threat processing, highlighting the anterior pulvinar and MD role in the acquisition of the CS-US association and suggesting that these regions underlie a parallel conscious vs. unconscious learning proposed by LeDoux and colleagues(LeDoux and Pine, 2016), Fig. 7a.

Our findings demonstrate that different pulvinar divisions hierarchically contribute to the integration of CS-US association in the anterior pulvinar through bottom-up processing. Although the anatomical projections within pulvinar divisions are not well characterized, our functional data-driven approach revealed a critical role for the medial pulvinar as a hub region facilitating communication among pulvinar divisions. Specifically, our trial-wise analysis showed that the medial, inferior, and lateral pulvinar process threat-related information earlier than the anterior pulvinar. The anterior pulvinar exhibited activation in response to CS+ starting at trial 2, whereas activation in the other pulvinar divisions occurred as early as trial 1. Network modeling further identified the medial pulvinar as a central hub, facilitating communication with other divisions. Using a robust mediation model, we demonstrated that the medial pulvinar appears to mediate the relay of sensory CS+ information from the inferior and lateral pulvinar to the anterior division for higher-order integrative learning. This hierarchical model aligns with previous evidence suggesting that the inferior and lateral pulvinar are associated with processing basic sensory information(Berman and Wurtz, 2010, 2008; Bridge et al., 2015; Cortes et al., 2024). While the medial pulvinar, which receives projections from deep layers of the superior colliculus, supports more advanced functions such as attention and working memory(Bridge et al., 2015; Homman-Ludiye and Bourne, 2019). These findings provide novel insights into the functional specialization of pulvinar divisions during threat learning, suggesting feedforward functions in the inferior and lateral pulvinar and high-order integration in the anterior pulvinar.

The pulvinar divisions and MD are actively engaged in extinction learning, as indicated by increased activation in response to CS+ during the first two trials, followed by a diminished response. This decline in activation likely reflects a shift in the emotional valence of the CS+ to match that of the CS−, suggesting a rapid extinction process in the human thalamus. Despite the involvement in extinction, these nuclei remained sensitive to the extinguished threat during safe contextual cues in extinction recall and exhibited increased activation to the extinguished CS+ when the stimuli were presented within threat contextual cues during renewal. This pattern suggests engagement in retrieval suppression during recall, along with threat salience processing during both recall and renewal. The MD and pulvinar continue to monitor and evaluate the extinguished threat across contexts rather than relying on static safety memories. These nuclei appear to engage in dynamic evaluation, learning, and decision-making during future encounters with the extinguished CS+.

The classical Pavlovian model proposes that extinction learning forms a new safety memory, competing with the original threat memory acquired during conditioning(Bouton, 2002; Milad and Quirk, 2002). Contextual changes often favor either the safe or threat memory. Maren and colleagues(Maren et al., 2013; Maren and Quirk, 2004) described the hippocampal-prefrontal-amygdala model for contextual memory. The hippocampus projects to the basolateral amygdala, vmPFC, and dACC. Although the vmPFC supports safe memory recall by projecting to the intercalated cells (which inhibit the central nucleus of the amygdala), the dACC enhances threat memory renewal by projecting to the basolateral amygdala (which activates the central nucleus of this structure). Our findings suggest that the thalamic connectivity may influence the balance between recalling safe vs. threat memory by modulating the competitive interaction between the dACC and vmPFC. The increased MD-dACC connectivity during recall may reflect a thalamic-driven reconfiguration of prefrontal circuits, enabling the vmPFC to become more functionally dominant and promote safe memory expression. On the other hand, the anterior pulvinar-vmPFC connectivity during renewal may destabilize the influence of the vmPFC, allowing the dACC to regain dominance and facilitate threat memory retrieval. The thalamic connectivity, thus, appears to orchestrate a context-dependent functional balance between the dACC and vmPFC. This balance may serve as a neural “toggle switch” between safe vs. threat memory expression. We integrated this suggested flexible responding mechanism to changing environmental contexts in Maren’s circuit model of emotional memory, Fig. 7b.

The LGN contribution is consistent across all phases of threat learning, as evidenced by our trial-wise results, which pointed to increased activation in response to CS+ that diminished immediately after the first trial. This distinct pattern indicates readiness for imminent visual stimuli, regardless of the actual emotional valence, as conditioning, extinction, recall, and renewal elicited similar BOLD patterns. This aligns with previous studies that highlighted the LGN’s engagement in selective attention and anticipation of visual stimuli(Mahoney and Schmidt, 2024; O’Connor et al., 2002; Saalmann and Kastner, 2009). The LGN, as a first-order nucleus(Cortes et al., 2024), primarily supports a feedforward function, while the pulvinar, as part of the visual thalamus, is more closely associated with a broader functional processing(Cortes et al., 2024) including emotional valences of stimuli. This distinction underscores their joint but specialized contributions to adaptive threat responses.

Although distinct thalamic roles in threat learning have been proposed, fMRI data do not fully capture the complexity of this structure. Pulvinar divisions, MD, and LGN each contain different neuron subtypes and finer anatomical subdivisions, which may serve diverse functions. Future advancements, such as higher-resolution human brain atlases, could improve our ability to study these nuclei with better anatomical precision. Additionally, since different sensory modalities preferentially engage distinct thalamic nuclei, the specific thalamic roles we described may not be consistent across different experimental designs, particularly in studies using auditory threat learning. The pulvinar divisions’ relationships during conditioning are purely functional and might be supported by direct or indirect anatomical projections. Finally, given the indirect nature of fMRI data and the absence of direct brain signal manipulations, our findings should not be interpreted as evidence of causality. Further research is needed to examine causal mechanisms underlying dynamic neural representations of threat learning within the thalamus.

This study’s insights raise critical future questions at the intersection between neuroscience, psychology, and mental health, particularly regarding the neural mechanisms underlying associative threat learning. First, the crucial role of the anterior pulvinar and MD during the acquisition of the CS-US association sets the stage for developing more precise brain interventions, focusing on refining maladaptive fear reactions. Future studies could explore the optimal parameters for applying non-invasive techniques to inhibit these thalamic regions during or immediately after fear conditioning. This approach is particularly promising for clinical populations and individuals exposed to high-risk environments, such as emergency medical staff, firefighters, and paramedics.

Second, the distinct contributions of the MD-dACC and anterior pulvinar-vmPFC circuits to safe vs. threatening memory suggest potential intervention targets for prioritizing one memory over the other. Activating the MD-dACC may facilitate recalling safe memories while engaging the anterior pulvinar-vmPFC circuits might reinforce fear memory pathways. Experimental designs in controlled laboratory settings could target these circuits to identify specific parameters for enhancing safe memories. This avenue holds therapeutic potential, particularly in conditions such as aviophobia; activating a safe memory circuit before a flight might suppress fear relapse during the actual flight.

Third, the anterior pulvinar-MD relationships raise questions related to the wide range of human behaviors, focusing on understanding neural gateways between conscious and unconscious learning. Studying the dynamics of information flow across this promising pathway could deepen our understanding of how sensory and cognitive information transits between conscious and unconscious states and brings about behaviors. This research line may pave the way for innovative learning methods and reveal neurocognitive mechanisms underlying the acquisition of new information in humans.

Together, our findings and emerging research directions underscore the thalamic nuclei’s vital role as a hub for fear acquisition and memory processing, offering promising avenues for theoretical advancements and clinical applications. As the primary neural gateway to the human brain, the thalamus is the first station for all sensory information except olfactory inputs. Modulating its functions is potentially an impactful strategy to induce widespread functional changes across the brain and influence different mental and behavioral expressions.

## Materials and Methods

### Participants

We analyzed the fMRI data of 293–412 human subjects of both sexes, aged 18–70 years old (M = 32.17 ± 13.1). All participants were proficient in English, right-handed, and had normal or corrected-to-normal vision. The exclusion criteria included a history of seizures or significant head trauma, current substance abuse or dependence, metal implants, pregnancy, breastfeeding, or positive urine toxicology screen for drugs of abuse. We followed the latest version of Helsinki’s declaration, and all procedures were approved by the Partners HealthCare Institute Review Board of the Massachusetts General Hospital, Harvard Medical School. All subjects provided written informed consent before taking part in the study. Results from this dataset have been published elsewhere with a different focus(Marin et al., 2020; Wen et al., 2024, 2022). The current results are novel and have not been previously published.

### Experimental procedure

Participants underwent two-day sessions of a validated threat learning paradigm in the MRI scanner (Figs. 1a, 4a–6a); the experimental contexts consisted of visual scenes on a computer display. On day 1, participants underwent a Pavlovian conditioning phase in which a neutral stimulus in context A (e.g., red light in an office) was paired with a 500ms electric shock (US) with a partial reinforcement rate of 62.5%. Another neutral stimulus in context A (e.g., blue light in the same office) was also presented but never paired with the shock (CS−). Participants were guided to select a highly annoying but nonpainful shock level during a pre-experiment calibration stage, and we used that personalized level during conditioning. On the same day, we also conducted extinction learning in context B (e.g., red and blue lights in a casebook background). The stimuli were repeatedly presented with the removal of the expected reinforcement (i.e., no shock).

On day 2, the participants underwent two phases of a memory test. The first is extinction recall, including presenting the extinguished CS+ and CS− within safe contextual cues (context B used during extinction learning). The second is threat renewal, in which the stimuli were presented within threat contextual cues (context A used in conditioning). Both extinction recall and threat renewal included no US reinforcement. Across phases of threat learning, the duration of each trial was 6s, and the inter-trial intervals with a fixation screen ranged between 12 to 18 s (15 s on average). Finally, to control for potential confounds related to the experimental design, we pseudorandomized and counterbalanced the order of CS+ and CS− across phases and between subjects.

### MRI data acquisition and preprocessing

Two MRI settings were used to acquire the neuroimaging data. The first is a Trio 3T whole-body MRI scanner (Siemens Medical Systems, Iselin, New Jersey) using an 8-channel head coil. The functional data in this setting were acquired using a T2* weighted echo-planar pulse sequence with these parameters: TR = 3.0s, TE = 30 ms, slice number = 45, voxel size = 3 × 3 × 3 mm^3^. The second setting was also in the same scanner using a 32-channel head coil. The functional images were obtained using a T2* weighted echo-planar pulse sequence using TR = 2.56s TE = 30 ms, slice number = 48, voxel size = 3 × 3 × 3 mm^3^. The anatomical brain images were collected using a T1-weighted MP-RAGE pulse sequence, parcellated into 1 × 1 × 1 mm^3^ voxels. Elastic bands were affixed to the head coil device to reduce head motions.

Using the default pipeline in fMRIPrep, version 20.0.2(Esteban et al., 2020, 2019), we preprocessed the data and applied correction of slice timing, realignment of the functional images, and coregistration. In addition, the data was normalized into the Montreal Neurological Institute (MNI) space and smoothed with a 6-mm full-width half-maximum Gaussian kernel.

### Activation analyses

We applied the least-squares-based generalized linear model (GLM) for each participant to estimate the BOLD response to CS+ and CS− using SPM 12. We estimated the beta values for each voxel during each learning phase in the paradigm. Overall, the model included 32 regressors for the CS+ and CS−, a regressor for the context, and a regressor for shock in conditioning but not in other phases. The GLM also included six head movement parameters (x, y, z directions, and rotations). This first-level analysis resulted in contrast maps that we used to estimate the variability of these maps across all subjects at the group-level analysis. We then used the contrast maps from the group-level analysis to extract the averaged values across the voxels within predefined masks of the pulvinar divisions, MD, and LGN. These outputs were used to compare the BOLD response during the first four CS+ and four CS− trials. We averaged the activation for each CS type across trials to obtain the block-level activation.

The statistical analyses included t-tests, RM-ANOVA, network analyses, mediation models, and hierarchical regression models. All were conducted using JASP versions 0.18.3 and 0.19.3(JASP Team, 2024). False discovery rate (FDR) correction was applied across analyses. RM-ANOVA was used to analyze trial-by-trial BOLD responses as the same participants were measured across the trials. Assumptions of sphericity were checked, and Greenhouse-Geisser corrections were applied where necessary.

### Connectivity analyses

We computed the connectivity values using the CONN functional connectivity toolbox, version 22. a, for the MathWorks MATLAB program(Nieto-Castanon and Whitfield-Gabrieli, 2022; Whitfield-Gabrieli and Nieto-Castanon, 2012). We first segmented the brain images into different tissues of gray matter, white matter, and CSF. Then, we applied the standard denoising pipeline in the CONN toolbox(Nieto-Castanon, 2020) to the functional images to control the effect of potential confounding parameters, using a component-based noise correction method. Finally, bandpass frequency filtering of the BOLD time series between 0.008 Hz and 0.09 Hz was also applied.

We evaluated differences in connectivity between CS+ and CS− conditions using generalized psychophysiological interaction (gPPI) analyses. We defined the first eight trials of each condition as a block. The pulvinar divisions, MD, and LGN were defined as individual seed regions, and connectivity was assessed with target regions, including the dACC, sgACC, vmPFC, amygdala, and hippocampus. The model included seed BOLD signals as physiological factors, boxcar signals characterizing the task conditions convolved with an SPM canonical hemodynamic response function as psychological factors, and the product of the two as psychophysiological interaction terms. Functional connectivity changes were quantified using Fisher-transformed correlation coefficients of the psychophysiological interaction terms. At the group level, we used a GLM to assess task-related connectivity changes across participants. Differences in connectivity between conditions were evaluated using paired t-tests, applying false discovery rate (FDR) correction at p < 0.05. 10,000 bootstrap resamples were used to obtain BCa CI, providing more robust estimates of the connectivity mean differences.

### Masks

We defined the pulvinar divisions, MD, and LGN nuclei using predefined masks based on the neuroanatomical guidelines of the Automated Anatomical Labelling Atlas(Rolls et al., 2020). We also applied the same atlas guidelines to determine the masks for the two other anatomical regions; the amygdala and hippocampus. The masks for the functional regions were created using Neurosynth(Yarkoni et al., 2011) and the keyword ‘conditioning.’ For each region, we created 8mm spheres around the following identified peak coordinates: vmPFC (MNIxyz = −2, 46, −10), sgACC (MNIxyz = 0, 26, −12), and dACC (MNIxyz = 0, 14, 28).
